# A Distributed NFV-Enabled Edge Cloud Architecture for ICN-Based Disaster Management Services

**DOI:** 10.3390/s18124136

**Published:** 2018-11-26

**Authors:** Van-Ca Nguyen, Ngoc-Thanh Dinh, Younghan Kim

**Affiliations:** School of Electronic Engineering, Soongsil University, Seoul 06978, Korea; canguyen@dcn.ssu.ac.kr (V.-C.N.); thanhdcn@dcn.ssu.ac.kr (N.-T.D.)

**Keywords:** information-centric networking, edge computing, disaster management services, NFV, distributed cloud

## Abstract

In disaster management services, the dynamic binding between roles and individuals for creating response teams across multiple organizations to act during a disaster recovery time period is an important task. Existing studies have shown that IP-based or traditional telephony solutions are not well-suited to deal with such group communication. Research has also shown the advantages of leveraging information centric networking (ICN) in providing essential communication in disaster management services. However, present studies use a centralized networking architecture for disaster management, in which disaster information is gathered and processed at a centralized management center before incident responses are made and warning messages are sent out. The centralized design can be inefficient in terms of scalability and communication. The reason is that when the network is very large (i.e., country level), the management for disaster services becomes very complicated, with a large number of organizations and offices. Disaster data are required to be transmitted over a long path before reaching the central management center. As a result, the transmission overhead and delay are high. Especially when the network is fragmented and network connectivity from a disaster-affected region to the central management center is disconnected, the service may be corrupted. In this paper, we designed and implemented a distributed edge cloud architecture based on ICN and network function virtualization (NFV) to address the above issues. In the proposed architecture, disaster management functions with predefined disaster templates were implemented at edge clouds closed to local regions to reduce the communication overhead and increase the service availability. The real implementation and performance evaluation showed that the proposed architecture achieves a significant improvement in terms of average bandwidth utilization, disaster notification delivery latency, routing convergence time, and successful request ratio compared to the existing approaches.

## 1. Introduction

In recent years, the outstanding growth of the Internet and the introduction of many new applications have given rise to concerns about networking architecture to support scalable content distribution, security, mobility, and so on. Inspired by the fact that communication is used to disseminate information rather than to connect two end hosts, information-centric networking (ICN) has emerged as a promising architecture for future networks [1,2,3,4]. By naming information at the network layer, ICN enables features such as topology independent name-based routing, multicast/anycast, in-network caching, mobility support, and packet level security. ICN communication is typically driven by a pull-based (interest/data) mechanism and push-based (publish/subscribe) mechanism to facilitate the efficient and timely delivery of information to users; the advantages of ICN make it a good fit to disseminate information in disaster management services [5], which is considered an important service for smart cities.

Some studies [6,7] and content-oriented notification services (CNS) [5] have demonstrated the limitations of the current IP-based approaches and shown the benefits of ICN to provide essential communication for efficiently managing disasters based on the requirements of disaster communication [8]. Based on ICN, CNS [5] designed a flexible namespace that can represent the context (or organization) of the normal administrative hierarchy, as well as the special recipient hierarchy needed for incident response. CNS helps disaster service managers to mobilize and build team groups who will deal with the disaster scenarios. In addition, a dynamic binding between the roles and identity of users is also introduced to identities without burdening individuals to manually maintain the mapping. Thus, when people are trying to provide information, they can reach the proper receivers more easily. To enable such a feature, the authors define disaster templates for different kinds of disasters to automate disaster response planning at a centralized disaster management center. The authors also highlight that a government should prepare a disaster template beforehand. With the introduction of disaster templates, CNS is implemented as a content-oriented notification service using a centralized architecture for disaster management.

However, the implementation of CNS has the limitations of a centralized architecture. Centralized architecture for disaster management is inefficient in terms of communication overhead, scalability, and fault tolerance. When the network is very large, e.g., country level, the management for disaster services becomes very complicated, with a large number of organizations and offices. Using a centralized networking architecture, disaster information (disaster report, disaster sensor tracking information, commands, etc.) is required to be transmitted over a long path before reaching the central management center. As a result, the transmission overhead and delay are high. Especially when the network is fragmented and network connectivity from a disaster-affected region to the central management center is disconnected, the service may be corrupted.

In this paper, we designed and implemented a distributed edge cloud architecture based on ICN and network function virtualization (NFV) [9] to address the above issues. In the proposed architecture, disaster management functions with predefined templates were implemented at edge clouds closed to local regions to reduce the communication overhead and increase service availability in cases of fragmented network scenarios during disasters. Intuitively, the geographically distributed computing model can help to reduce communication costs (i.e., bandwidth consumption) and the latency for disaster notification delivery using nearby computation and storage resources. Moreover, it can also improve privacy depending on the characteristics of a specific area. Name-based binding and forwarding advantages of ICN as well as predefined templates are used to enable disaster information to be forwarded to correct corresponding receivers without requiring connectivity to the central management office. To enable the collaboration between regions for a disaster response, we also defined a new namespace to support new features for disaster management systems and enable communication between the central disaster management center (C-DMC) and edge disaster management centers (E-DMCs) (i.e., at local regions), as well as among E-DMCs. A predefined adjacent location template is designed to help an E-DMC in identifying the appropriate support E-DMC, which helps to mobilize support teams to deal with incidents. The architecture is implemented using network function virtualization (NFV) technology [9] and software-defined networking (SDN) [10,11] to facilitate the deployment and management, which are also discussed in our previous works [12,13]. The real implementation and performance evaluation showed that the proposed architecture achieves a significant improvement in terms of average bandwidth utilization, disaster notification delivery latency, routing convergence time, and successful request ratio compared to the existing approaches.

In summary, the proposed distributed architecture makes the following improvements compared to CNS, the state-of-the-art centralized architecture. The detailed results and explanations are presented in the Evaluation Section.

- The proposed model achieves better bandwidth utilization with lower communication overhead and higher scalability compared to CNS.
- The proposed model achieves lower disaster notification delivery latency compared to CNS.
- The proposed model achieves shorter routing convergence time for disaster name template setup.
- The proposed model can work in fragmented networks when the connection to the central management office is disconnected. By contrast, CNS requires the connection reachability to the central management office.
- The proposed model achieves higher service availability compared to CNS.

The remaining sections are organized as follows: Section 2 presents the related studies. Subsequently, we introduce our proposed architecture and system design, such as protocols and naming schema to support communication between the system components in Section 3. In Section 4, we show our implementation and evaluation results by leveraging NFV technology.

## 2. Related Work

### 2.1. Disaster Management Services

The high frequency of major disasters that have occurred in the past decade have resulted in a growing attention and investment on the national and regional disaster response methods, as well as disaster management services. Those disaster management systems have the requirements of communicating effectively, interoperating, and coordinating among different teams and agencies for disaster relief operation. Communication methods have been considered the key factor in any disaster management system for coordinating and interoperating among diverse organizations [14]. With no effective communication approaches, it is difficult for various organizations (i.e., first responders, police, commanders, hospitals, citizens, etc.) to interoperate for effective disaster management services. On scene, first responders and remote supporting agencies have no ability to update situational awareness, resource management, personnel coordination, or unifying commands. Flexibility is also an important requirement for a disaster management system. The reason is that disaster zone environments can change rapidly, and responders may need to adjust the capabilities to match the needs and situations. Roles, especially, in the disaster management hierarchy may change dynamically.

In the literature, there are extensive studies on communication networking in extreme environments. The studies investigate disruption tolerant networking [15,16], wireless sensor networks for physical condition and disaster monitoring [17,18], mobile networking solutions [19], etc. ICT capabilities have to enable first responders to communicate effectively. The current legacy infrastructure for disaster management services is mainly based on circuit telephony networks for emergency calls. Therefore, the current system is limited to support data communication and interactive services, such as contextual content, written messages, or video content. Recently, next-generation emergency services (NG112, NG9-1-1) [20,21,22] have been proposed to attempt to design and implement enhanced emergency services using the IP infrastructure. These will enable authorities and citizens to communicate for emergency services with the data communication technologies that citizens use in their daily life. For example, several studies deal with location identification, which maps the location of victims to services such as LoST (i.e., connecting victims with the closest police station) [20,21,23].

### 2.2. The Limitations of IP-Based Solutions and the Potential of ICN for Disaster Management Services

However, the current services are only designed for emergency calls from citizens. In addition, the architectural design is based on the TCP/IP infrastructure, which has the limitations as described in CCN [24]. In fragmented disaster scenarios, the design cannot work because it relies heavily on end-to-end communication. The services will be out of reach when the servers are unavailable.

Information-centric networking (ICN) shifts the focus of communication from the location to the identity of the contents. ICN architectures leverage in-network caching, location-independent, and name-based routing for the efficient distribution of contents over the network [1,2]. Content-centric networks (CCN) [24] are one of the most attractive ICN research projects. The content name in CCN is using a hierarchical name, which is encoded with the content identifier, version number, segment information, data digest, and publishers. The goals of CCN are to accommodate a network architecture with a secure, flexible, and scalable network, thereby addressing the requirements for secure content distribution on a massive scale to a differing set of end devices. CCN secures individual pieces of content rather than securing the connection. Using names instead of IP addresses to help CCN can provide flexible communication. Additionally, distributed caches with named and secured contents are automatically populated on demand or selectively prepopulated. When an Interest message requests a content, CCN delivers the content matching with the Interest to the user from the nearest cache, passing through fewer network hops. In CCN, routing is performed using similar algorithms to current IP infrastructure, for example, Internet link-state IGPs (IS-IS or OSPF). Moreover, CCN allows the aggregation of similar names in name resolution and data routing information.

In COPSS [25], the existing CCN approaches are enhanced with a push-based multicast capability. COPSS is designed to facilitate a highly dynamic and large-scale publish/subscribe system and is able to deliver content in a timely manner. COPSS uses the concept of hierarchical content descriptors (CDs) to name components of information disseminated. A piece of content can have multiple CDs and a CD can identify multiple content items. CDs are utilized to subscribe to and publish information by subscribers and publishers, respectively. A consumer interested in a CD can subscribe to the CD and will receive all the contents of the CD and its descendants. Publishers do not need to have knowledge about intended recipients. An ICN router in COPSS is equipped with a subscription table (ST). The ST is used to maintain a list of downstream subscribers with the outgoing faces towards the subscribers. The subscribe packet is forwarded towards the rendezvous node (RN) using the forwarding table entry at a COPSS aware router. Along the path, the COPSS aware routers add this entry in their ST. In COPSS routers, subscriber information can be aggregated, and the incoming interfaces are included in the list of subscribers downstream. The received data messages which match with CDs in the ST will be forwarded to the list of interfaces that have subscribers downstream. The ST is implemented as a Bloom-filter [26], which tests all incoming data and will be sent to the faces whose bloom-filter contains the CD(s) the packet satisfies. This allows a publish packet from a publisher with the corresponding CD to reach subscribers.

In disaster scenarios, we should consider handling the requirements for communication [8] in both fragmented and nonfragmented cases. In case of a fragmented network, delay/disruption tolerant networking (DTN) [27] is a promising ad-hoc communication method. DTN provides network services continuously via a network that has no specific infrastructure. Communication in DTNs has to tolerate much larger delays in message delivery than in usual networks. The authors of [7] introduced NREP, a mobile name-based system. In NREP, message replication is limited by time and space within a certain geographic area and with specific life expectancy based on the type of service, e.g., SOS, warning, chat. In Reference [6], the proposed application facilitates the integration of multiple transfer methods, e.g., pull (interest/data) as well as push (publish/subscribe), for content delivery. Every ICN node (including producer and consumer end-points, as well as intermediate routers) can select the most appropriate transfer method to deliver each content item. Levering in-network storage is an inherent capacity of ICN. Content may be also transferred on a store-and-forward basis, with storage nodes acting as gateways between different transfer methods for named objects.

## 3. Disaster Scenario Descriptions

### 3.1. Use Cases

Disaster scenarios can be divided into three categories: Adversarial, natural, and accidental disaster. For example, when an accidental incident, such as a fire, occurs at a place in a town or earthquake/flooding occurs in a region, sensor events are reported or an emergency call is dialed to the disaster management center to request the officers and ambulances to deal with the incident. When the emergency notification is sent to the disaster management center located in the local region (i.e., at the town or the local district), the response from the local disaster management office will be better and more efficient than if the report is sent from the central disaster management center placed at any place in a country. Because the distributed disaster management center is placed nearby users, it helps to reduce end-to-end communication latency and has a better capability to deal with failures which can occur along transmission paths.

In the case of many incidents occurring in a local region, the local officers may not have the ability to handle all the incidents. The edge disaster management center will send requests for support from adjacent officers to the central disaster management center. The central disaster management center will find an appropriate support team of another edge cloud. The mobilized support team from the adjacent location will subscribe to receive the incident information.

Furthermore, when the connection between the central and the edge disaster management centers (i.e., due to disaster) is disconnected, edge disaster management centers can work independently for the local area. The request for support to deal with incidents will be spread over all available connections. The earliest response will be accepted to support the incidents.

### 3.2. Requirements of the System

Currently, services are deployed using cloud computing technology, such as centralized data-centers (core-clouds) and distributed data-centers (edge-clouds). The centralized data-center model has become a popular platform for data computing. Centralized data-centers comprise the thousands or hundreds of thousands of servers. Although this model has been proven quite successful in deploying and scaling cloud services, it has drawbacks. For instance, the failure of a region can lead to a service dropout. This is an issue as some applications and services are sensitive to latency. A distributed cloud, which is geographically distributed, and smaller data centers can be a solution to this situation. A distributed cloud offering nearby computation and storage resources not only can reduce communication overheads, costs, and latencies, but it can also improve the local privacies. The deployment of edge cloud using NFV technology can reduce the complexity of the management system. When an NF failure is affected by other incidents or any hardware or software reasons, the service operator can easily create NF backups to enable services to guarantee the quality of the services or to load-balance traffic with a minimum latency.

Our proposed architecture is motivated from CNS and inherits the features of CNS to serve disaster services in local areas in order to achieve timely responses and efficient communication. In addition, for some requirements for communication in a local area, such as predefined roles, dynamically formed groups, group communication, content hierarchy, recipient hierarchy, and priority-based communication, the proposed architecture should also support the communication between C-DMC and E-DMCs or between E-DMCs together. In cases where the connection between E-DMCs and C-DMC are disconnected, data mules are mobilized to help the network to exchange data.

## 4. The Proposed Architecture

This section presents the proposed architecture. The list of acronyms used in this section is given in Table 1.

### 4.1. System Architecture

Our goal is to build a hierarchical disaster management system dealing with disaster scenarios in a distributed manner. This system can provide a service with a timely response, efficient communication, and information dissemination. Distributed edge cloud computing using NFV is a promising solution for cost reduction, deployment agility, and scalability in terms of failure and high traffic volume.

We implement disaster services in an NFV environment using an orchestration system (MANO) at the core cloud. The MANO creates VNFs at the edge cloud, which implements disaster services. The disaster management center is responsible for mobilizing officer teams dealing with incidents by instantiating a new subscribed namespace for mobilized teams. With the centralized management model, the centralized center will manage and mobilize all places at country level.

**Figure 1:**
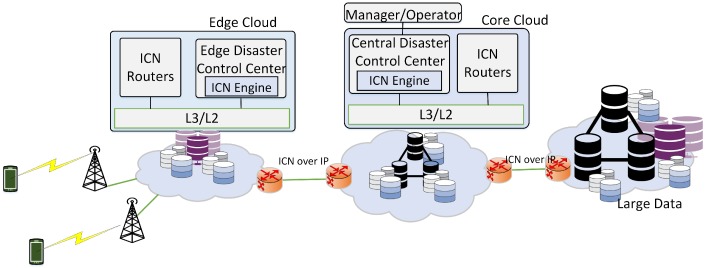
High-level view of information-centric networking (ICN) based disaster service platform.

Figure 1 illustrates the high-level view of the disaster service platform, which is deployed following a distributed controller model on core-cloud and edge-clouds.

The components of disaster services include three main parts:

- **ICN routers**: ICN routers are connected to implement an ICN forwarding plane for disaster services. ICN routers forward and store data packets in the network.
- **Central Disaster Management Center (C-DMC)**: The C-DMC manages disaster service in local areas. The C-DMC manages and updates the control information for E-DMCs, such as the template of subscribing namespaces to deal with incidents and hierarchical administrators. The C-DMC is a coordination point to coordinate between E-DMCs when an E-DMC requests the support from another area to deal with disaster situations. It also disseminates disaster warning messages from the government to E-DMCs.
- **Edge Disaster Management Centers (E-DMCs)**: E-DMCs manage namespace templates and hierarchical administrators, which is used to name the information for communication between users and between disaster managers and users in the local area. E-DMCs receive emergency notifications from the mobilize officer teams and instantiate the template of namespaces and hierarchical administrators in publish/subscribe communication to exchange disaster information between teams. Local E-DMC communicates with the C-DMC to register, update control information, send the supporting requests from the C-DMC or other E-DMCs, and receive and then spread disaster warnings to users.

The C-DMC and E-DMCs communicate with each other through edge ICN routers. In CNS [5], the centralized disaster management center stores and manages the rules to instantiate roles for individual members and namespace to deal with incidents in all areas. This method would increase the complexity of the management system, especially in large-scale areas, and increase response delay in disaster scenarios. The distributed hierarchical architecture is proposed, aiming to reduce the communication overhead and enable timely responses. In the proposed architecture, each E-DMC is an edge disaster management center for a local area and the C-DMC is a centralized management center for all E-DMCs. In cases of disconnected connections between tge C-DMC and E-DMCs due to the effects of disasters or any failure, E-DMCs still work independently.

### 4.2. Naming Schema

In ICN, the naming scheme can be adapted for specific applications and contexts [1,2,3,4]. For disaster management services, CNS [5] designs an efficient naming schema for supporting both communication within the normal administrative hierarchy and cross-functional communication in disaster scenarios. The naming schema also provides an effective and flexible binding between the roles and individuals, which is a good fit for disaster management services. In addition, the proposed distributed architecture in this paper is compared with CNS, the centralized architecture. To produce comparable results with CNS, we used the naming schema in CNS as the reference naming schema. We modified and adapted the schema to fit into our model.

#### 4.2.1. Naming Schema for Local Communication

The naming schema in CNS [5] is modified and adapted for communication in the local edge clouds. The namespace for subscription is managed by local disaster management centers. Based on the concept of CNS, we extended the disaster template namespace to manage the new namespace “Emergency/Support”.

In some cases, disaster management for a local area may require support from other nearby areas. As a result, a local E-DMC may request support from other E-DMCs. The support teams will be named “supporting” instead of with a unit in the district or town which is managed by the local E-DMC.

**Figure 2:**
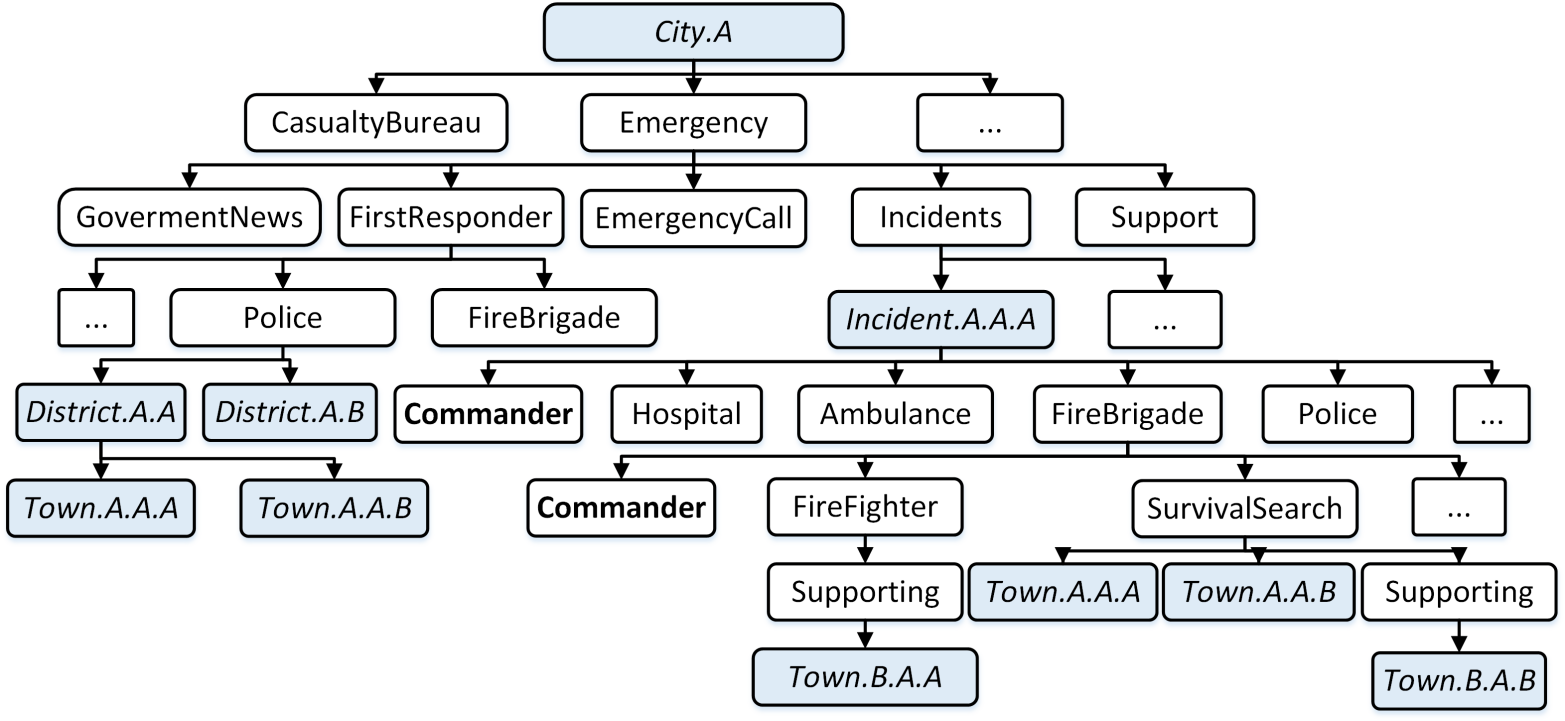
Namespace in requested edge disaster management center.

Figure 2 presents an example of the namespace and administrative hierarchy at local “city A” to deal with “incident.A.A.A”. The namespace is set up for role-based teams who are mobilized to deal with “incident.A.A.A” and the support teams “town.B.A.A” and “townB.A.B” from other areas. The support team is identified by the name “supporting”.

In the support E-DMC, which is sent the support teams to deal with the incidents, the support teams will be managed “emergency/support”, which is created by the request from C-DMC, with the name of the incident as support, and the roles of the support teams.

**Figure 3:**
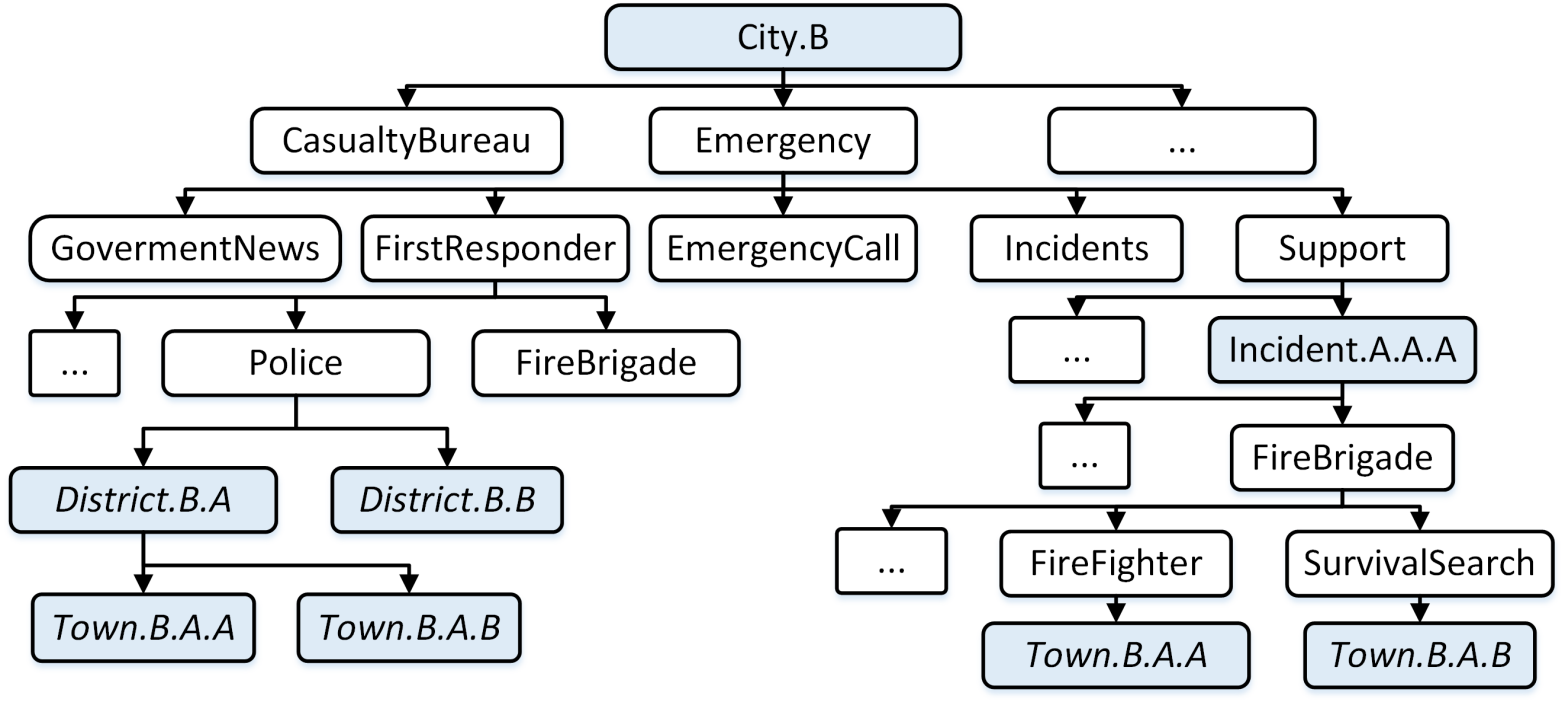
Namespace in support edge disaster management center.

Figure 3 presents an example of the support namespace and administrative hierarchy at city B. The namespace is set up for the team’s town B.A.A and town B.A.B, who are mobilized to support the incident A.A.A of city A. It can be identified by the name: “support”.

#### 4.2.2. Data Structure of Central Disaster Management Center

The C-DMC stores all administrative hierarchy, incidents, and support information of the disaster service system. The C-DMC and E-DMCs exchange updated disaster control information.

**Figure 4:**
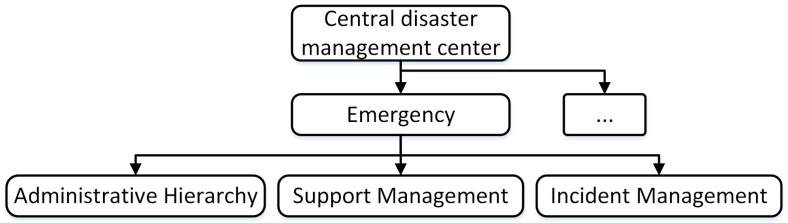
Data structure of central disaster management center.

Figure 4 shows the data structure of the C-DMC, including the hierarchical administrator, the managing incidents, and the supporting management. In our context, we consider the request support for the first responder team, e.g, police, firefighter, hospital, ambulance.

**Figure 5:**
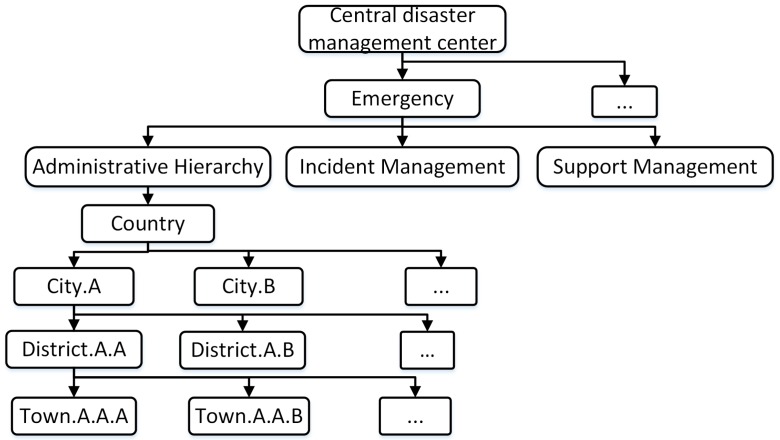
Data structure of hierarchical administrator.

Administrative management information identifies the exact geographical location and represents the administrative hierarchy units. Figure 5 presents an example of hierarchical administrators at the country level. It includes the geographical administrative units following town, district, and city (province) level. It helps to identify the exact location where an incident occurs to mobilize the team to deal with the incident.

Incident management information identifies the incidents in each E-DMC. It is organized based on namespace to subscribe the incident information in CNS [5].

**Figure 6:**
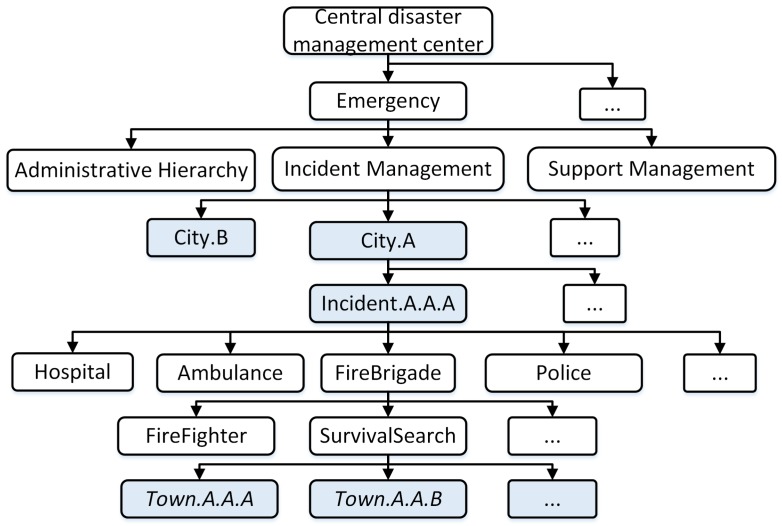
Data structure of hierarchical incident management.

Figure 6 shows an example of incident management with the namespace required to set up for role-based teams who deal with incidents. It can be classified based on the assigned name of E-DMCs and the incident names in each E-DMC. For example, the edge-cloud at city A (the name is the assigned same with the location) manages “incident.A.A.A” with information about the mobilized local team “town.A.A.B”, who is handling the incident.

Support management information helps to identify a support team who is supporting to deal with an incident at another E-DMC.

**Figure 7:**
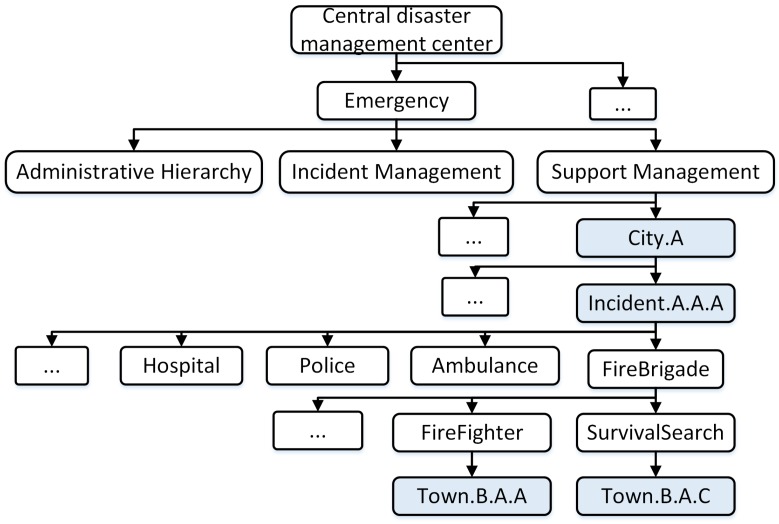
Data structure of hierarchical support management.

Figure 7 presents an example of supporting management with the namespace required to set up for role-based teams who are managed by an E-DMC and go to support the incident managed by another E-DMC. It can be classified based on the assigned name of the E-DMCs and the incident names in each E-DMC. For example, the incident A.A.A of city A is supported by the fire brigade teams “town.B.A.A” and “town.B.A.C” from city B.

**Figure 8:**
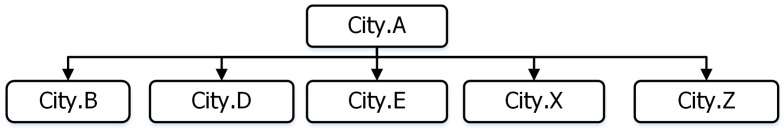
Adjacent location template city (province) layer.

**Figure 9:**
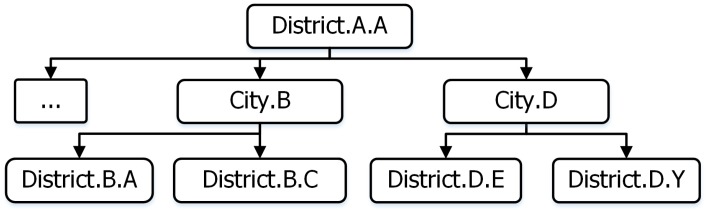
Adjacent location template district layer.

**Figure 10:**
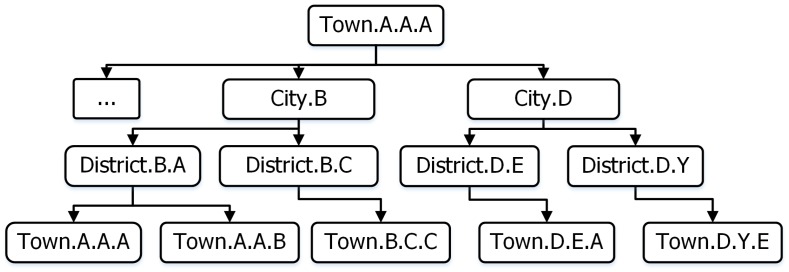
Adjacent location template town layer.

In order to find the most appropriate support team to deal with the incident happening at a region of an E-DMC, we introduced an adjacent location template to help the C-DMC. Figure 8, Figure 9 and Figure 10 present an example of adjacent location templates of the city level, district level, and town level, namely, city A, district A.A, and town A.A.A, respectively.

The adjacent location template presents the geographical relationship among all the places which are managed by another E-DMC.

#### 4.2.3. Naming Schema for Intercloud Communication

The naming schema is used for communication between the C-DMC and E-DMCs or among edge clouds. The central controller C-DMC is assigned the name “central”. The namespace design assists E-DMCs to exchange disaster control information, such as precisely determining the controller who receives a message, the type of services, what the request action and when the request is sent. It also supports aggregated subscriptions/queries in order to reduce the subscription request load.

The naming schema for communication is designed as follows:

*/cloud-id/type-of-service/type-of-action/time*

The component *cloud-id* identifies the receiver C-DMC and E-DMCs. The parameter *type-of-service* determines the kind of service using the information in the context. For example, “emergency” for a disaster scenario is considered for an emergency context. The component *type-of-action* determines the function used to process the message, e.g., register, update control information. or request support. The *time* component identifies the time of the support request, e.g., T:6:00—6 a.m. For example, we have “central/emergency/request/T:06:00”. Sometimes, many requests are sent to request support from the C-DMC or other E-DMCs, so duplication can occur. The *time* parameter is used to determine the supporter needs to mobilize support teams to support that incident.

### 4.3. Intercloud Protocols

One of the advantages of our proposed architecture is the scalability of the system and the ability to work independently when the connection with the C-DMC is disconnected. This subsection describes our design for intercloud protocols for the interactions between E-DMCs and the C-DMC, and among E-DMCs, to enable collaborations among different regions for disaster response.

**Figure 11:**
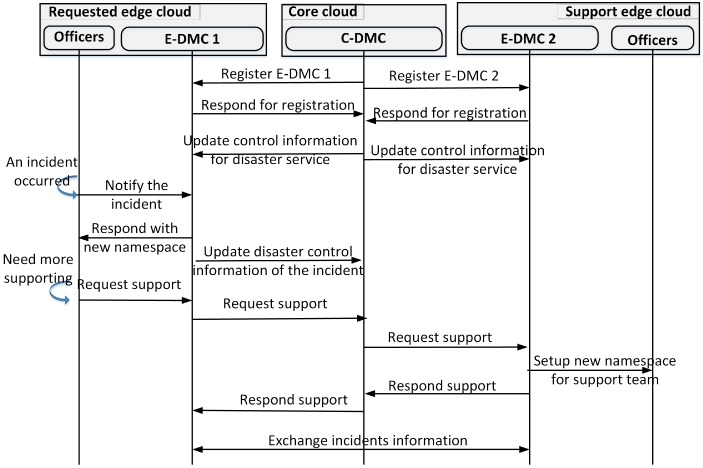
Exchange control information between edge disaster management centers (E-DMCs) and the central disaster management center (C-DMC).

Figure 11 presents a mechanism to setup and exchange the control information for disaster services between E-DMCs and the C-DMC. Firstly, the C-DMC registers the E-DMCs which are used to serve disaster services. The C-DMC will manage all information about incidents. The C-DMC distributes the control information, such as a template of subscribing namespaces and hierarchical administrators to deal with incidents. When an incident occurs, an emergency notification is sent to the local E-DMC. Based on the template namespace, the local E-DMC sends an order to instantiate the new subscribe namespace for the teams who are chosen to deal with the incident.

In cases where the local team does not have enough ability to handle incidents, E-DMCs will send a support request to the C-DMC. The C-DMC then calculates to find a potential E-DMC which can support the incident where the managing E-DMC’s request originated from. The support E-DMC mobilizes and instantiates the namespace for the support teams to deal with the incident. The C-DMC sends the response with the supporting E-DMC information to the requesting E-DMC. The requesting E-DMC and support E-DMC begin to exchange disaster information by adding the assigned name of E-DMCs.

**Figure 12:**
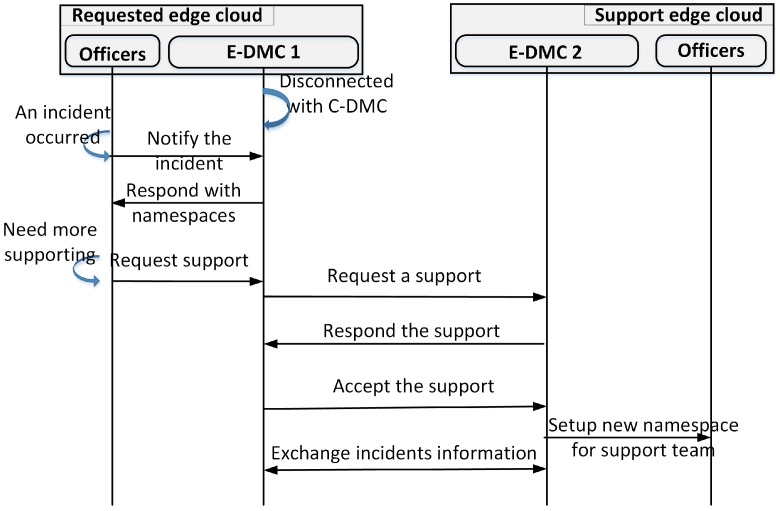
Exchange control information between E-DMCs.

Figure 12 presents the procedure in cases where the connection between the C-DMC and E-DMCs is disconnected. In such cases, the support request will be spread to the adjacent E-DMCs. The receiving E-DMCs will process and respond with support to the requesting E-DMC. The requesting E-DMC receives the responses and accepts the first response to support the incidents. An accepted message is sent to the first E-DMC response. This E-DMC will instantiate the namespace for support teams to go to deal with the incident.

## 5. Experimental Result and Evaluation

### 5.1. Implementation and Configurations

**Figure 13:**
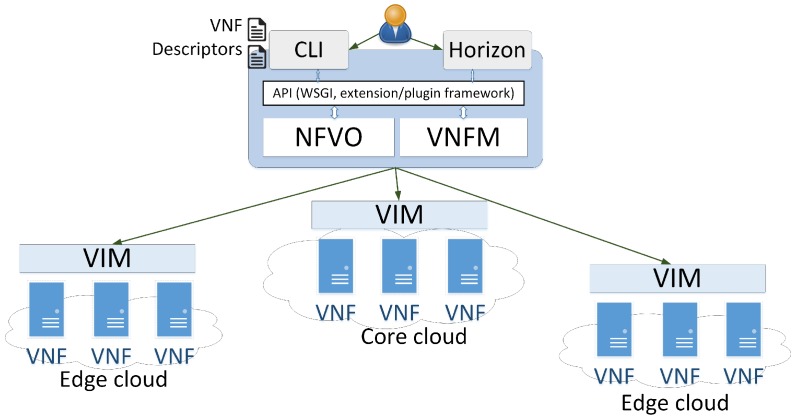
The implementation of the architecture in the network function virtualization (NFV) environment.

**Figure 14:**
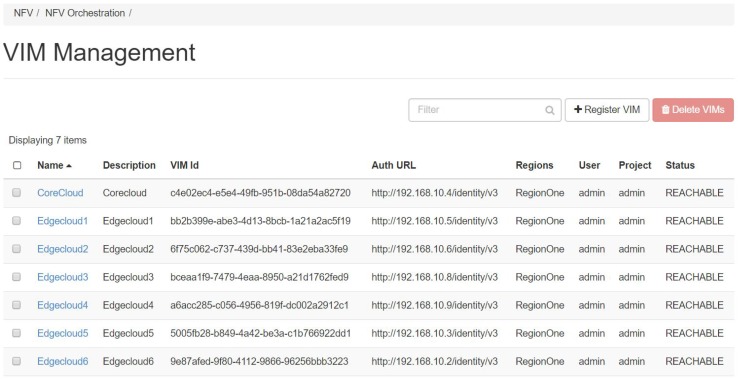
The management dashboard of the architecture in the core cloud.

In this part, we describe the implementation details of our proposed system architecture in the NFV environment. The distributed model with E-DMCs and the C-DMC was implemented with OpenStack [28] as NFVI and Tacker [29] as NFVO and VNFM. This implementation followed the ETSI NFV reference model [9]. The testbed deployment used multiple OpenStack installations (multi-VIM) [28]. The detailed implementation of the architecture is shown in Figure 13. Figure 14 shows the management dashboard of the architecture in the core cloud. In the beginning, a service manager used Tacker to register the multiple OpenStack installations (multi-VIM), which were to be used to deploy the disaster services. Following the ESTI NFV model, we exploited Tacker to manage the VNF life cycle and orchestrated them to create, as well as configure, ICN forwarders which implement the ICN forwarding plane to forward ICN messages to users. For that, we used TOSCA templates [13,28] for Tacker, which included information about network configurations of ICN forwarders to automate the ICN node and ICN forwarder deployment. The ICN forwarders were deployed using the VPP plugin [30]. The ICN forwarders were connected to local disaster management centers. ICN forwarders, E-DMCs (i.e., local disaster management centers), and the C-DMC (i.e., the central disaster management center) were deployed as VNFs. The detailed configurations for experiments are presented in Table 2.

The proposed distributed model was compared with CNS, the state-of-the-art centralized model, and Mobile-IP [31] as the IP-based model. For comparable evaluations, we used the same data set with CNS [5]. Other parameters were inherited from CNS [5]. The results for bandwidth utilization, disaster notification delivery latency, and average routing convergence time were obtained through testbed experiments with fully connected networks. The results for the ratio of successful support requests under fragmented network scenarios were obtained through simulations. Experiments were deployed with physical servers using Intel core i7 with CPU of 3.4 Ghz, 32 GB of RAM, 500 GB of HDD, and Ubuntu 16.04 LTS version. VNFs were created using the Pike version of Openstack and Tacker. For the disaster template deployment, we used the SDN controller of OpenDaylight Carbon version with 8 GB of RAM and 40 GB of HDD. The detailed implementations of a VNF can be found in our previous studies [12,13].

### 5.2. Bandwidth Utilization

**Figure 15:**
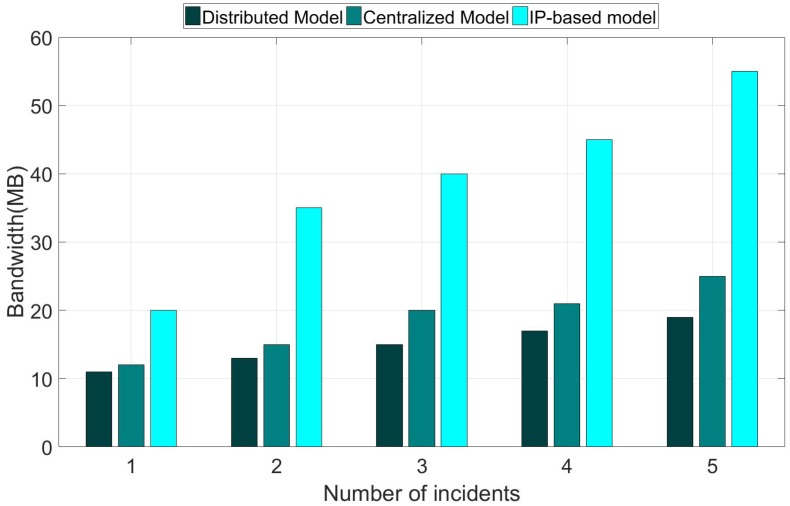
The comparison of bandwidth utilization between models.

An important requirement of a disaster management system is the efficiency of spreading disaster information to the users. For that reason, we measured the utilization of bandwidth for an experiment for spreading disaster information to all users in the network [5]. Figure 15 shows the bandwidth utilization when the warning messages are spread by the disaster management center. The figure shows that the proposed distributed model and the centralized model (i.e., CNS), which use the information-centric networking approach for disaster information dissemination, witnessed a considerable bandwidth saving compared to the IP-based model. In addition, the proposed distributed model consumed a significant lower bandwidth compared to the centralized model. Moreover, the bandwidth consumption of the distributed model increased only slightly when the number of incidents increased. By contrast, the bandwidth consumption of the centralized model increased significantly and the bandwidth consumption of the IP-based model increased rapidly regarding the growing number of the incidents. This result implicitly indicates that the distributed model provides a higher scalability compared to the centralized model and the IP-based model. The reasons behind the improving achievement of the proposed distributed model are as follow. First, the proposed model requires that only one copy of the message be sent from the sender to each link receiver through the recipient hierarchy by taking the advantage of the ICN-based multicast mechanism [2]. As the message is information-centric based and addressed, receivers can fetch the message from any cache or intermediate node. In the IP-based approach, messages are sent in an end-to-end communication manner, so a message may be sent multiple times to a link in different nodes. In the case of the IP-based approach, the end-host cannot fetch the content from a neighboring node who also received the same data. In the IP-based approach, the disaster information is disseminated, mainly relying on unicast, which results in higher traffic consumption and higher latency, as shown in the next subsection. Second, subscriptions for disaster notification can be aggregated based on the name prefix to reduce the number of forwarding operations. Third, the message is not required to be sent from and through the central management office like in the centralized model, because the edge (E-DMC) at a local network can process the disaster information dissemination to its local network, thus saving significant bandwidth consumption and forwarding latency for the message, especially the bandwidth of links to the core network. The above reasons can also be used to explain the results discussed below.

We can see that the bandwidth utilization of ICN-based disaster services is more effective than that of IP-based disaster services. The reason is that the ICN-based method takes advantage of the multicast mechanism to spread the warning messages.

### 5.3. Disaster Notification Delivery Latency

**Figure 16:**
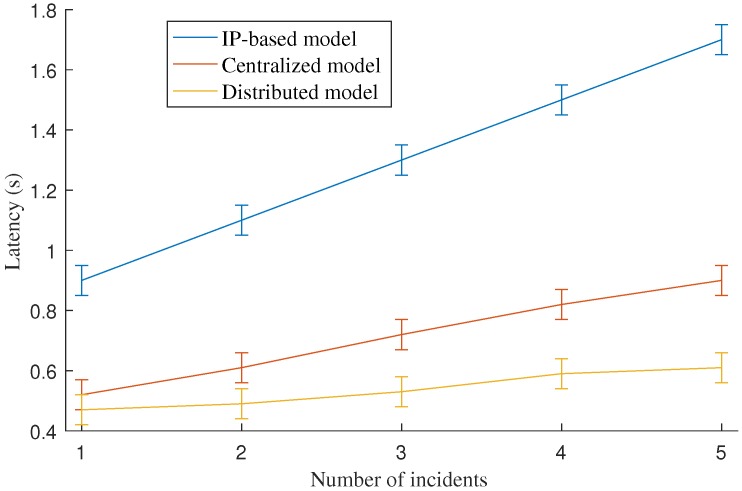
The comparison of average disaster notification delivery latency.

Figure 16 shows the average latency for the disaster notification delivery latency of the proposed distributed model in comparison with the centralized model and the IP-based model. The figure shows that the proposed distributed model achieves the lowest latency for disaster notification delivery latency. Moreover, the latency increases only slightly when the number of incidents increases. By contrast, the latency of the centralized model increases significantly and the latency of the IP-based model increases rapidly regarding the growing number of the incidents. As a result, the improvement achievement of the proposed model is proportional to the number of incidents, which indicates the scalability of the proposed model. For example, in the case of one incident, the latency improvement ratio of the proposed distributed model is 9.8% compared to the centralized model. The improvement ratio is increased to 32% in the case of five incidents.

The trend of latency results is similar to that of the bandwidth consumption shown in Figure 15.

### 5.4. Average Routing Convergence Time for Disaster Name Template Setup

In Reference [5], the communication groups are created dynamically and the naming schema follows the predefined template in specific disaster scenarios. Using pure ICN, the control information is distributed, and the router propagates and builds a forwarding table by itself. When an incident occurs, a user makes a report to the disaster management center. After receiving the report, manager sends a request for the local police to confirm the incident. Then, the manager receives the confirmation from the local police. The manager forms some support group to support the incident, and the new subscribe information is sent to the support group. Officers who received the new subscribe information will subscribe to a new content descriptor (CD) to receive information related with the incident. The routers will distribute routing information to build a forwarding table. The main processing times include the delay of confirmation from the local police and routing convergence times for building a forwarding table of routers. On the other hand, NFV-based routers are virtual routers and they are often managed by control planes and centralized management planes. When we need more capacity In ICN-based disaster services, the most important thing is the naming schema and the deployment of naming schemas. Leveraging the advantage of the management plane in NFV, it is easy to configure and manage the naming schema through a centralized management plane. Instead of each officer needing to subscribe to content and routers needing to exchange routing information for building forwarding table, all routers can be configured by the control plane following the order of the management plane.

In this part, we compare the average routing convergence time for the setup of a disaster name template between the CNS with traditional name template dissemination and the proposed distributed model with SDN/NFV. In the former, after the disaster name template is issued by the central office, the disaster name template will be disseminated over the network. In the latter, the disaster name template is set up using the SDN approach to directly install the disaster name template to specific network regions. We used OpenDaylight as an SDN controller to distribute the disaster name template and forwarding rules to routers.

**Figure 17:**
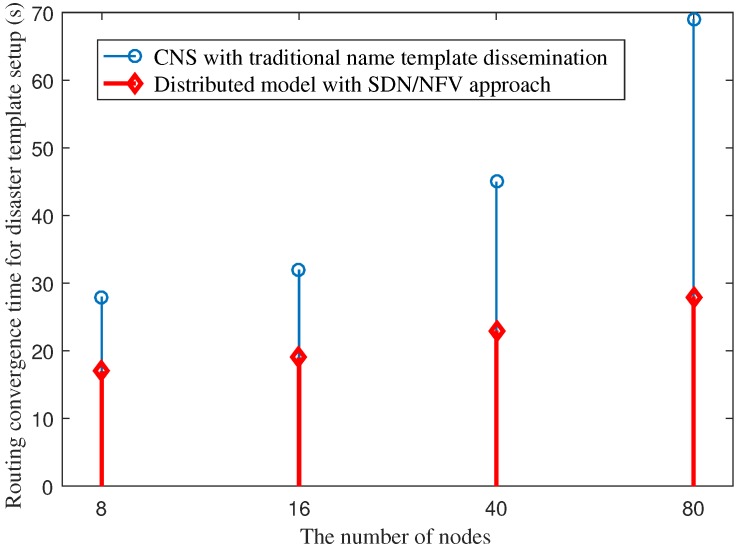
Average routing convergence time for disaster name template setup under various numbers of NDN nodes in the network.

Figure 17 presents the comparison of the average routing convergence time between the two models. The figure indicates that our implementation using the SDN approach for the disaster name template setup achieves a significantly shorter routing convergence time. As the disaster name template is installed directly by the SDN controller through specific setup paths, instead of broadcasting over the network for routing information propagation as in CNS, the convergence time increases only slightly when we increase the number of NDN nodes. The convergence time of CNS increases rapidly in proportion to the number of nodes.

### 5.5. The Ratio of Successful Support Requests under Fragmented Network Scenarios

**Figure 18:**
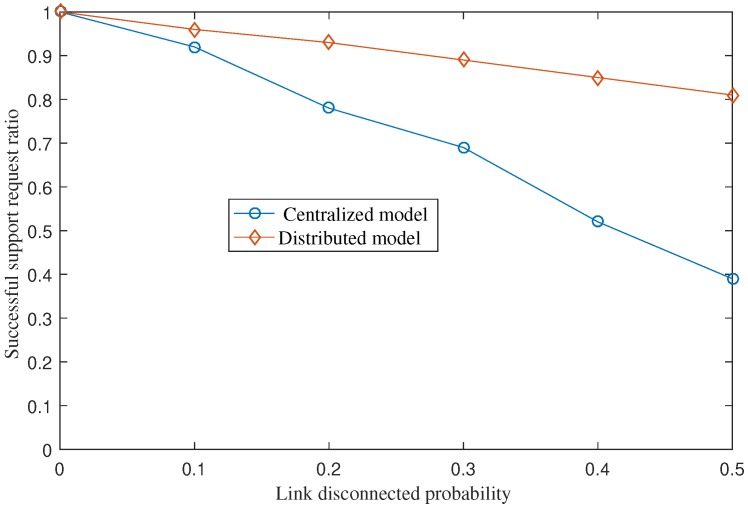
Average ratio of successful support requests under various link disconnected probability values. Note that the link disconnected probability indicates the probability that a link in the network is disconnected.

With the proposed distributed architecture, the disaster services can still run even when regions are disconnected from the C-DMC (i.e., due to disasters, etc.). In other words, the disaster services can still run locally at a region even when its E-DMC is disconnected from the C-DMC. In addition, with the proposed intercloud communication protocol, the proposed scheme enables an E-DMC to process sending requests for support from neighboring E-DMCs successfully without requiring reachability to the C-DMC. In this section, we conduct a simple analysis simulation to show the advantage of the distributed architecture in fragmented network scenarios. In the simulation, the disconnected probability of each link varies from 0% to 50%. Note that the link disconnected probability indicates the probability that a link in the network is disconnected. Disasters are assumed to happen at a random edge (i.e., a random region). We assume the region with a disaster will need support from nearby neighboring regions. Therefore, it will send a support request for help. A support request is successful if the requesting region receives at least one response from other regions and the name space setup for the support team is completed. We obtained the average percentage of successful support requests by CNS and the proposed distributed architecture. The results are presented in Figure 18. The figure shows that the proposed distributed model achieves 82% of successful support requests even when the link disconnected probability is 0.5. The result for the centralized model is 39%. The results indicate that the distributed model works much better in fragmented network scenarios. A request in the centralized model may fail due to the following reasons: (1) The requesting region is disconnected from the central; (2) the support team which is assigned to support another region is disconnected from the central; (3) the requesting region is disconnected from the support team. In the case of the distributed model, a support request fails if the requesting E-DMC is disconnected from all neighboring edges. The request–support process in the distributed model can be processed in a peer-to-peer approach among E-DMCs without the need of the C-DMC. As a result, the disaster management service availability of the distributed model is improved compared to CNS.

## 6. Conclusions

This paper presented a distributed NFV-based architecture for disaster management services to address the high overhead, long delay, and low service availability limitations of the current ICN-based centralized architecture. The distributed architecture was implemented with testbeds based on ICN and NFV to exploit the benefits of edge computing for disaster management services. The experimental and analysis results showed that the proposed architecture achieves a significant improvement in terms of communication overhead and disaster notification delivery latency compared to CNS, the centralized model, and the IP-based model, and in terms of routing convergence time for disaster name template setup and service availability compared to CNS. The distributed model has a limitation of high deployment cost. In particular, the model requires a deployment of the disaster management service at edge cloud in each region (i.e., a city or a district). For future studies, we plan to investigate deployment schemes to optimize the deployment cost for the distributed model, to add interactive features for first responders and victims in mobility scenarios, and to integrate the model with disaster monitoring sensor networks, like existing sensor-cloud systems [32,33,34,35,36]. 

## Figures and Tables

**Table 1:** List of Acronyms.

Acronym	Meaning
ICN	information-centric networking
CNS	content-oriented notification service
MANO	network functions virtualization management and orchestration
NFV	network function virtualization
VNF	virtual network function
SDN	software-defined networking
E-DMC	edge disaster management center
C-DMC	central disaster management center
CC2420	a RF transceiver type for sensors

**Table 2:** The detailed configurations for experiments.

Entity	Parameters	Version
Physical server	CPU: Intel Core i7 @3.4 Ghz, RAM: 32 GB, HDD: 500 GB	
OpenStack	Master version	Pike
Tacker	Master version	Pike
Operating system	64 bits	Ubuntu 16.04 LTS
SDN controller	RAM: 8 GB, HDD: 40 GB	OpenDaylight Carbon
vForwarder	RAM: 3 GB, HDD: 40 GB	Ubuntu 16.04 LTS
